# High Serum CEA and CYFRA21-1 Levels after a Two-Cycle Adjuvant Chemotherapy for NSCLC: Possible Poor Prognostic Factors

**DOI:** 10.7497/j.issn.2095-3941.2012.04.009

**Published:** 2012-12

**Authors:** Xue-feng Lin, Xiao-dong Wang, Da-qiang Sun, Zhi Li, Yue Bai

**Affiliations:** 1 Tianjin Medical College, Tianjin 300222, China; 2 Department of Thoracic Surgery, Tianjin Chest Hospital, Tianjin 300051, China

**Keywords:** non-small-cell lung cancer, carcinoembryonic antigen, cytokeratin 19 fragement, adjuvant chemotherapy, prognosis

## Abstract

**Objective:**

The aim of this study was to test whether carcinoembryonic antigen (CEA) and cytokeratin 19 fragments (CYFRA21-1) can be used as a prognostic factor for non-small-cell lung cancer (NSCLC) after two cycles of adjuvant chemotherapy in NSCLC patients.

**Methods:**

A total of 169 patients underwent at least two cycles of adjuvant chemotherapy. The serum levels of CEA and CYFRA21-1 were recorded after the second cycle of chemotherapy, and the patient follow-up was conducted. Overall survival (OS) and disease-free survival (DFS) were used as the primary endpoint and the secondary endpoint, respectively.

**Results:**

The high levels of CEA and CYFRA21-1 after two cycles of adjuvant chemotherapy were poor prognostic factors for OS, with risk ratios (RR) of 2.003 and 1.702, respectively. A high CEA level was a poor prognostic factor (RR 1.152) for DFS. The median survival time (MST) of the high CEA level group was 26 months, whereas that of the normal group was 61 months (*P*<0.0001). The median DFS time of the high CEA group and the normal group was 34 and 53 months, respectively (*P*<0.0001). The MST of the high CYFRA21-1 group and the normal group was 43 and 56 months, respectively (*P*<0.0001).

**Conclusions:**

The high serum levels of CEA or CYFRA21-1 after two cycles of adjuvant chemotherapy are poor prognostic factors for NSCLC patients.

## Introduction

Adjuvant cisplatin-based chemotherapy improves disease-free survival (DFS) and overall survival (OS) in patients with resected stages IB-IIIA of non-small-cell lung cancer (NSCLC). However, after resection, the prognosis of NSCLC patients who underwent adjuvant chemotherapy varied because of individual characteristics. Tumor markers (TM) may be used as prognostic factors for NSCLC. Although several new biomarkers have drawn increasing interest in NSCLC, carcinoembryonic antigen (CEA) and cytokeratin 19 fragments (CYFRA21-1) continue to play important functions owing to their universality, low price, and repeatability. Currently, no single TM exists for improved diagnostic specificity. Thus, only 30% to 70% of lung adenocarcinoma patients exhibit elevated serum CEA levels^[^^1^^]^. No cancer diagnosis is based solely on high TM level; that is, TM has a limited diagnostic value. At present, serum TMs are mainly used to estimate the stage of the disease^[^^1^^,^^2^^]^, treatment response^[^^3^^]^, and prognosis^[^^4^^]^. This study aimed to explore whether the serum level of CEA and CYFRA21-1 can be a prognostic factor after two cycles of adjuvant chemotherapy for NSCLC patients who received complete resection.

## Materials and Methods

### Clinical data

This retrospective study collected 169 NSCLC cases from Tianjin Chest Hospital between July 2005 and December 2007. These patients underwent complete resection and two cycles of adjuvant chemotherapy. All cases that met the following requirements were selected simultaneously: *i*) complete resection (International Committee of Lung Cancer Staging 2005); *ii*) all cases were confirmed by abdominal computed tomography (CT), brain CT or magnetic resonance imaging (MRI), bone electrochemotherapy (ECT), and other tests to exclude distant metastasis; *iii*) all cases were in stages IB to IIIA (pathological tumor–node–metastasis); *iv*) all cases received adjuvant chemotherapy within two months after operations and cycles of adjuvant chemotherapy ≥2. The exclusion criteria of cases include the following: *i*) malignancy history, including lung cancer; *ii*) neoadjuvant chemotherapy or radiotherapy before surgery; and *iii*) radiotherapy after surgery.

### Specimen collection and measurement

Serum CEA and CYFRA21-1 levels were tested after two cycles of adjuvant chemotherapy. Roche cobas 6000 Elecsys was used for the test, with kits provided by Roche. The normal range of TMs was <4.7 ng/mL for CEA and <3.3 ng/mL for CYFRA21-1. TM levels higher than the upper limit of the normal were considered high (abnormal).

### Treatment

Platinum combined with gemcitabine/paclitaxel/vinorelbine was administered. Drug combinations were given as follows: GP (gemticabine, 1000 mg/m^2^ to 1250 mg/m^2^, d1, d8; cisplatin, 75 mg/m^2^, d1 or carboplatin AUC=5, d1); TP (paclitaxel 175 mg/m^2^ to 200 mg/m^2^, d1; cisplatin 75 mg/m^2^, d1 or carboplatin AUC=5, d1); and NP (vinorelbine 25 mg/m^2^, d1, d8; carboplatin 80 mg/m^2^, d1). At least two chemotherapy cycles were conducted.

### Follow-up

Follow-ups were conducted among patients who finished at least two cycles of adjuvant chemotherapy: once every three months in the first year post-surgery, once every six months in the second year, and once every year until five years later. The follow-ups were mainly carried out in the outpatient service; otherwise, the patient follow-up was conducted by telephone. Outpatient follow-up consisted of physical examination, chest CT scan, and imaging of related body parts in symptomatic, such as bone ECT, brain CT scan/MRI, and abdominal type B ultrasound/CT. The intention-to-treat principle was applied in every patient.

This study was mainly concerned with prognosis. The primary endpoint was OS or the time from surgery date to death date. If the patient was still alive until the last follow-up, the survival time ended by the last follow-up, and the data were censored. The second endpoint was DFS, which was cut off by the time of distant or regional recurrence and death without recurrence (censored).

### Statistical analysis

The Cox proportional hazards regression model was used to analyze the prognosis influenced by multiple factors, and the Kaplan-Meier method was used for survival analysis. Survival between groups was compared using the log-rank test. Censored data were removed in the OS and DFS analyses to improve interpretation. Data were entered and analyzed with SPSS version 13.0. Median follow-up was estimated with inverse Kaplan–Meier method^[^^5^^]^. *P*<0.05 was considered significant.

## Results

The overall follow-up period was from 3 months to 66 months, and the median follow-up period covered 53 months. A total of 15 patients (8.88%) were lost to follow-up. Univariate and multivariate analyses were performed. The factors included age, gender, histological type, T stage, N stage, CEA level, and CYFRA21-1 level. The results are shown in **Table 1**.

**Table 1:** Clinical characteristics and *P* values of OS and DFS analyzed by single-factor analysis.

Characteristics	*n* (%)	*P* (OS)	*P* (DFS)
Age, years		0.561	0.465
Median	63		
Range	32-78		
Gender (%)		0.995	0.809
Male	116 (68.6)		
Female	53 (31.4)		
Histological type		0.839	0.941
Adenocarcinoma	63 (37.3)		
Squamous-cell carcinoma	83 (49.1)		
Mixed	23 (13.6)		
T stage		0.161	0.187
T_1_ (T_1a_/T_1b_)	18/26		
T_2_ (T_2a_/T_2b_)	80/22		
T_3_	23		
N stage		<0.0001	<0.0001
N_0_	59 (34.9)		
N_1_	57 (33.7)		
N_2_	53 (31.4)		
CEA		<0.0001	<0.0001
Normal	98 (58.0)		
High	71 (42.0)		
CYFRA21-1		<0.0001	0.002
Normal	106 (62.7)		
High	63 (37.3)		
Drug option		0.328	0.136
GP	54 (32.0)		
TP	92 (54.4)		
NP	23 (13.6)		

The Cox proportional hazards regression model suggested that N stage, CEA level, and CYFRA21-1 level were independent prognostic factors for OS, as shown in **Table 2**. N stage and CEA level were prognostic factors for DFS, as shown in **Table 3**.

**Table 2:** Multivariate analysis by Cox for OS.

Variables	β	SE (β)	Wald χ**^2^**	*P*	RR	95%CI for RR
N stage	0.343	0.099	12.038	0.001	1.409	(1.161, 1.711)
CEA	0.695	0.178	15.260	<0.0001	2.003	(1.414, 2.838)
CYFRA21-1	0.532	0.184	8.391	0.004	1.702	(1.188, 2.439)

**Table 3:** Multivariate analysis by Cox for DFS.

Variables	β	SE (β)	Wald **χ^2^**	*P*	RR	95%CI for RR
N stage	0.514	0.115	19.855	<0.0001	1.672	(1.334, 2.097)
CEA	0.594	0.192	9.591	0.02	1.812	(1.244, 2.639)

**Figure 1:**
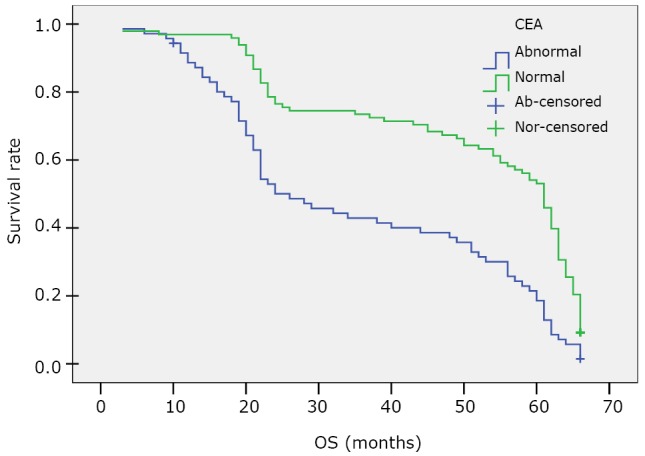
Kaplan-Meier survival curve of OS for the high (abnormal, ≥4.7 ng/mL) and normal CEA level (<4.7 ng/mL) patients.

The last follow-up date was July 31, 2011. A total of 118 patients (69.8%) had regional or distant recurrence, and 107 patients (63.3%) died of lung cancer. Kaplan-Meier analysis suggested that the median survival time (MST) of the 169 patients was 53 months (95% CI; 46.651, 59.349) and the 5-year survival rate was 35.9%. Comparison of OS between the high (abnormal) serum CEA level group (CEA ≥4.7 ng/mL) and the normal group (CEA <4.7 ng/mL) is presented in **Table 4** and **Figure 1**. The MST of patients with high serum CEA level was 26 months, whereas that of patients with normal CEA level was 61 months. The 5-year survival rate of patients with high CEA level was 18.0%, significantly lower than that of those with normal CEA level (50.2%, *P*<0.0001).

**Table 4:** Comparison of OS between normal CEA (<4.7 ng/mL) and high CEA level (≥4.7 ng/mL) groups by log-rank test*.

Group	*n*	Death	Censored in the first 2 years (%)	Censored between the 3^rd^ and 5^th^ year (%)	MST (95%CI)	2-year rate, %	5-year rate, %
High	71	59	2 (2.82)	3 (8.33)	26.00 (19.071, 32.929)	49.3	18.0
Normal	98	48	3 (3.06)	7 (9.33)	61.00 (58.583, 63.417)	75.8	50.2
Overall	169	107	5 (2.96)	10 (9.01)	53.00 (46.651, 59.349)	64.6	35.9

*log-rank, **χ^2^**=15.013, *P*<0.0001

**Figure 2:**
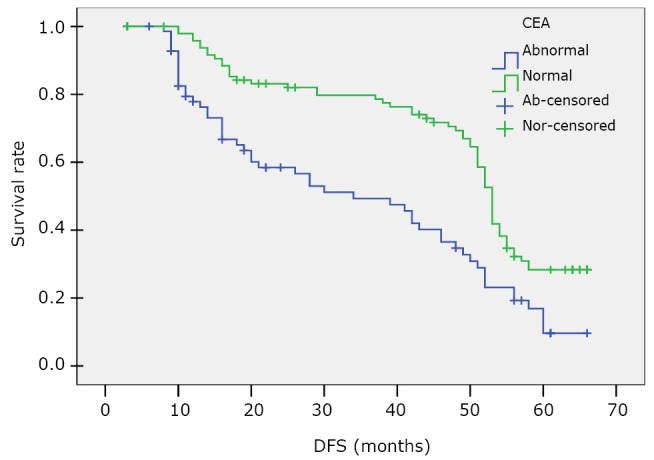
Kaplan-Meier survival curve of DFS for the high (abnormal, ≥4.7 ng/mL) and normal CEA level (<4.7 ng/mL) patients.

The median DFS of patients with a high CEA level was 34 months, significantly shorter than that of those with a normal CEA level (53 months, *P*<0.0001). The results are presented in **Table 5** and **Figure 2**.

**Table 5:** Comparison of DFS between normal CEA (< 4.7 ng/mL) and high CEA level (≥ 4.7 ng/mL) groups by log-rank test*.

Group	Events^†^	Censored in the first 2 years (%)	Censored between the 3^rd^ and 5^th^ year (%)	Median DFS (95%CI)	2-year rate, %	5-year rate, %
High	61	2 (2.82)	3 (8.33)	34.00 (20.234, 47.766)	41.2	14.3
Normal	57	3 (3.06)	7 (9.33)	53.00 (51.751, 54.249)	63.5	47.9

*log-rank, **χ^2^**=26.211, *P*<0.0001. ^†^The number of patients who were found regional or distant recurrence.

**Figure 3:**
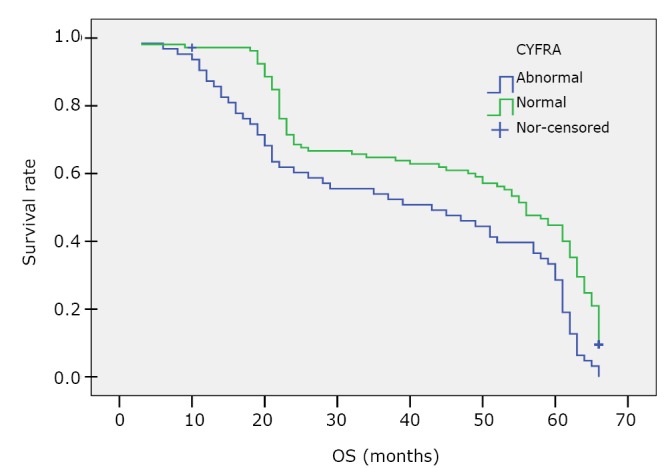
Kaplan-Meier survival curve of DFS for the high (abnormal, ≥3.3 ng/mL) and normal CYFRA21-1 level (<3.3 ng/mL) patients.

Comparison of the OS between the high (abnormal) serum CYFRA21-1 level group (CYFRA21-1 ≥3.3 ng/mL) and the normal level group (CYFRA21-1 <3.3 ng/mL) is shown in **Table 6** and **Figure 3**. The MST of patients with high CYFRA21-1 level was significantly shorter than that of those with normal CYFRA21-1 level (43 months *vs.* 56 months, *P*<0.0001).

**Table 6:** Comparison of OS between normal CYFRA21-1 (< 3.3 ng/mL) and high CYFRA21-1 level (≥ 3.3 ng/mL) groups by log-rank test*

Group	*n*	Death	Censored in the first 2 years (%)	Censored between the 3^rd^ and 5^th^ year (%)	MST (95%CI)	2-year rate, %	5-year rate, %
High	63	50	3 (4.76)	2 (5.56)	43 (20.778, 65.222)	60.0	14.1
Normal	106	57	2 (1.89)	8 (10.67)	56 (50.530, 61.470)	68.3	43.8

*log-rank, χ**^2^**=14.700, *P*<0.0001.

## Discussion

Arriagade et al.^[^^6^^]^ reported in a randomized controlled trial that NSCLC patients who received postoperative platinum-based adjuvant chemotherapy exhibited a significant 4.1% absolute increase in the 5-year survival rate (44.5% *vs.* 40.4%). However, not all patients who underwent adjuvant chemotherapy can achieve the desired effect, and sometimes they even had poor prognosis. This single-center retrospective analysis showed that the 5-year survival rate of high serum CEA or CYFRA21-1 level group was poorer than that of the normal level group after a two-cycle adjuvant platinum-based chemotherapy. We established a Cox regression model with age, gender, histological type, T stage, N stage, CEA level, CYFRA21-1 level, and drug option as variables. The results showed N stage, CEA level, and CYFRA21-1 level are prognostic factors for OS, whereas N stage and CEA level are prognostic factors for DFS. As N stage escalated, OS and DFS became poorer in this study. N stage is one of the main prognostic factors used in routine clinical practice for NSCLC patients^[^^7^^,^^8^^]^.

Studies on TMs presently focus on stage prediction, curative effect, and prognosis. Kawachi et al.^[^^2^^]^ divided the preoperative CEA level into normal group (≤5 ng/mL), high group (≤30 ng/mL), and very high group (>30 ng/mL). The results suggest that the preoperative CEA level was a highly positive predictor of the pathologic stage. Ishiguro et al.^[^^3^^]^ retrospectively analyzed 24 NSCLC patients with high serum CEA levels (>5 ng/mL) and received chemotherapy followed by surgery. Serum CEA levels after chemotherapy significantly decreased in patients who achieved partial response; by contrast, no significant difference was observed in the CEA levels of patients with either stable disease or no response to chemotherapy. A number of studies^[^^9^^,^^10^^]^ also suggested that serum CYFRA21-1 levels constituted the prognostic factors for advanced NSCLC patients receiving chemotherapy. A meta-analysis^[^^11^^]^ which included 2,063 NSCLC patients, suggested that high serum CYFRA21-1 level before treatment was a poor prognostic factor.

In conclusion, this study suggests that either elevated serum CEA level or elevated serum CYFRA21-1 level after a two-cycle adjuvant chemotherapy following complete resection is a poor prognostic factor.
